# Family members' knowledge, attitudes, practices, and caregiver burden in managing the health of patients with severe burn injuries

**DOI:** 10.3389/fpubh.2025.1450356

**Published:** 2025-05-19

**Authors:** Huili Wang, Jiaming Yang, Mengjia Xia, Linying Zhu, Mirong Liao

**Affiliations:** Taizhou Hospital of Zhejiang Province Affiliated to Wenzhou Medical University, Linhai, Zhejiang, China

**Keywords:** knowledge, attitude, practice, burden, family members, severe burn injuries

## Abstract

**Background:**

Caregivers, particularly family members, play a vital role in the treatment and recovery of burn patients, yet their psychological burden and care competency are often overlooked. Despite their crucial role, there is limited research examining their knowledge, attitudes, and practices in burn care management.

**Objective:**

This study aimed to investigate knowledge, attitudes, practices (KAP), and burdens experienced by family members in managing the health of patients with severe burn injuries.

**Methods:**

A cross-sectional study was conducted at Zhejiang Province, from November 1, 2023, to January 31, 2024. Data were collected using a self-administered KAP questionnaire and Zarit Caregiver Burden Scale 12-item Short Form.

**Results:**

The study enrolled 459 valid participants, of which 253 (55.12%) were female. Median scores [25th percentile, 75th percentile] for knowledge, attitude, practice, and caregiver burden were 12 [9, 14], 17 [16, 19], 33 [31, 37], and 16 [10, 23], respectively. Correlation analysis revealed that higher caregiver knowledge was associated with improved attitudes (*r* = 0.35, *P* < 0.001) and more proactive practices (*r* = 0.36, *P* < 0.001), while being inversely related to caregiver burden (*r* = −0.18, *P* < 0.001). Better attitudes strongly predicted enhanced practices (*r* = 0.75, *P* < 0.001) and reduced caregiver burden (*r* = −0.24, *P* < 0.001). Similarly, more proactive practices were associated with lower caregiver burden (*r* = −0.25, *P* < 0.001). Multivariate logistic regression analysis identified that income levels of 5,001–10,000 Yuan (OR = 12.6, 95% CI: [2.57, 62.4], *P* = 0.002) and 10,001–20,000 Yuan (OR = 8.05, 95% CI: [1.53, 42.3], *P* = 0.014) were found to be independently associated with increased caregiver burden.

**Conclusion:**

The study demonstrates that while family members generally hold positive attitudes and are proactive in the care management of patients with severe burn injuries, their knowledge levels remain suboptimal, and they experience a mild to moderate caregiver burden. Structured caregiver education programs focusing on long-term burn complications and psychological support should be implemented to enhance care quality and reduce caregiver burden.

## Introduction

Annually, 11 million burn injuries occur worldwide, with ~180,000 proving fatal, underscoring the global burden of this affliction (https://who.int/en/news-room/fact-sheets/detail/burns). Severe burns, defined as burns involving more than 20% of the Total Body Surface Area (TBSA), represent one of the most serious forms of trauma and are particularly catastrophic due to the extensive destruction of skin barriers dysregulation of immune responses. This dysregulation triggers an extreme immune response causing systemic inflammation, wound expansion, and micro thrombosis, which, combined with compromised skin integrity, increases vulnerability to infectious complications (7–11).

With advancements in burn care, survival rates have improved, yet survivors often endure long-term physical and psychological consequences, affecting their quality of life, ability to return to work, and overall mortality (12). Severe burn injuries typically require intensive, prolonged care, and aim to restore the patient's functionality to as close to pre-injury status as possible, necessitating significant resources and specialized expertise throughout the treatment process (13, 14). The treatment and recovery of individuals with severe burns involve concerted efforts from both professionals and non-professionals, including family caregivers. These caregivers are crucial to the recovery process, providing not only medical but also financial, social, emotional, and spiritual support. Informal healthcare support plays a vital role in the treatment continuum (1, 5, 15). However, the demands of caregiving, particularly for severe conditions such as burns, can be substantial, often impacting the caregivers' own health and wellbeing, and thus affecting their quality of life (16).

## Background

The KAP (Knowledge, Attitude and Belief, Practice) theory posits that knowledge serves as the foundation for behavior change, while attitudes and beliefs act as the driving forces that facilitate this change (17). The KAP model has been successfully applied in burn care settings to evaluate and improve caregiver competencies. Previous studies have demonstrated that KAP-based interventions can effectively enhance burn care outcomes. For instance, research has shown that improved knowledge of burn first aid among caregivers leads to better immediate care practices and reduced complications (2). In the context of long-term burn management, the KAP model has been particularly valuable in addressing wound care practices and rehabilitation compliance (18). Studies have also indicated that caregivers with higher KAP scores demonstrate better adherence to treatment protocols and experience lower levels of care-related stress (3). The KAP model serves as the theoretical foundation of this study. It conceptualizes knowledge as the basis for informed decision-making, attitudes as the motivational component, and practices as the observable behaviors resulting from this progression. This framework guides the structure of our questionnaire and the interpretation of relationships among variables in our analysis. According to this framework, the process of human behavior change encompasses three sequential steps: acquiring knowledge, forming attitudes/beliefs, and subsequently developing practices/behaviors (19). It is important to note that acquiring knowledge alone does not automatically result in behavior change; rather, it must first alter perceptions, which in turn influence behavior through these new perceptions (20). Understanding the KAP of caregivers is crucial for identifying existing gaps and designing targeted educational and support interventions that enhance care outcomes. Moreover, assessing caregiver burden is essential to devise strategies that protect their wellbeing, ensuring that they can continue their caregiving responsibilities without adverse effects on their health. This research is particularly pertinent as family members often undertake a critical, yet frequently unsupported, role in the continuum of care, which significantly affects both the recovery of the patient and the quality of life of the caregiver.

## Purpose

The present study aims to fill this gap. Given the scarcity of research specifically addressing the KAP in the context of severe burn injuries, significant knowledge gaps remain. While previous studies have extensively documented professional caregivers' experiences and competencies in burn units (21), family caregivers' perspectives and challenges remain underexplored. Specifically, there is limited understanding of how family members acquire and apply burn care knowledge, develop care-related attitudes, and implement daily care practices in home settings. Furthermore, the relationship between family caregivers' KAP levels and their perceived burden has not been systematically investigated. Therefore, this study aims to investigate the knowledge, attitudes, practices, and burdens experienced by family members managing the health of patients with severe burn injuries. This research is pivotal for several reasons: First, it addresses a critical gap in understanding how family caregivers manage severe burn patients, which is essential for developing targeted support interventions. Second, by examining both KAP and caregiver burden simultaneously, it provides a comprehensive view of the challenges faced by family caregivers. Third, the findings can directly inform healthcare policy and practice, potentially leading to improved outcomes for both patients and their caregivers. To guide our analysis, this study addresses the following questions: (1) What are the current levels of knowledge, attitudes, and practices among family caregivers of patients with severe burn injuries? (2) How are these KAP components interrelated? (3) What is the relationship between KAP levels and caregiver burden? (4) What demographic and clinical factors influence these outcomes?

## Materials and methods

### Study design and participants

This descriptive cross-sectional study was conducted at Zhejiang Province from November 1, 2023, to January 31, 2024. Participants included family members of patients who had experienced severe burns within the past 3 years. This 3-year timeframe was specifically chosen to capture both acute and chronic caregiving challenges, as it encompasses the critical phases of burn recovery: initial treatment and rehabilitation (0–6 months), scar management and functional recovery (6–24 months), and long-term adaptation (24–36 months). This comprehensive temporal window allows for a more complete understanding of the evolving caregiver experience across different stages of burn recovery.

Inclusion criteria included family members of patients with severe burns within the past 3 years, being immediate family members of the patients, aged 18 years or above, and capable of providing informed consent. Exclusion criteria were recent participation in similar research studies, caregivers who themselves were patients with severe burn injuries within the past 3 years and incomplete questionnaire responses or responses with logical inconsistencies, and presence of cognitive impairment that could affect survey responses. This study followed the Strengthening the Reporting of Observational Studies in Epidemiology (STROBE) guidelines for cross-sectional studies (4).

### Sampling and recruitment

Data collection was conducted using a convenience sampling method. Initially, six tertiary hospitals in Taizhou, Zhejiang Province with dedicated burn departments or burn and wound repair units were identified. Three hospitals (Zhejiang Taizhou Hospital, Taizhou Rehabilitation Hospital, and Taizhou Central Hospital) agreed to participate in the study, while three declined due to low staff engagement. Consecutive sampling based on admission dates was used to recruit eligible participants until the minimum required sample size was achieved.

### Data collection procedure

Create online questionnaires with Questionnaire Star and generate e-questionnaires to access QR codes and links. Questionnaires were distributed in tertiary hospitals in Zhejiang Province with specialized burns. To uphold data integrity and reduce the risk of duplicate or fraudulent entries, the system restricts entries to one per IP address, mandating full completion of all questionnaire sections. Four professionally trained research assistants meticulously reviews each submission for completeness, internal coherence, and logical consistency, enforcing mandatory responses throughout. Additionally, questionnaires with completion times below 90 s or above 1,800 s were removed.

### Instruments

The questionnaire designed to assess caregivers of burn patients was carefully crafted based on relevant guidelines, specifically those pertaining to burn rehabilitation treatment, as well as a thorough review of existing literature (22, 23). Initially drafted, the instrument was further refined following detailed feedback from three experts in burn care. To ensure its reliability, a pilot study was conducted with 58 participants, resulting in a Cronbach's alpha reliability coefficient of 0.934 for the overall scale. Subscale coefficients were 0.889 for knowledge, 0.886 for attitude, and 0.934 for practice, indicating high internal consistency.

The finalized questionnaire is divided into four sections: demographics, knowledge, attitude, and practice (Appendix). The knowledge section consists of 14 questions, with correct answers scoring 2 points and incorrect or unclear responses scoring 1 point, leading to a potential score range of 14–28 points. The attitude section includes 5 questions; however, question A4 is not included in the scoring. This section uses a five-point Likert scale, where responses range from very positive (5 points) to very negative (1 point), allowing for a total score range of 4–20 points. The practice section, similarly, includes 8 questions utilizing a five-point Likert scale, with responses varying from always (5 points) to never (1 point), and a total score range of 8–40 points. Scores exceeding 70% of the maximum possible in each section are indicative of adequate knowledge, a positive attitude, and proactive practice behaviors (24). Additionally, the Zarit Caregiver Burden Scale 12-item Short Form was employed to evaluate the burden experienced by caregivers, The scores for each item are summed to obtain a total score, with a range of 0–88 (0–20: low or absence of burden; 21–40: mild to moderate burden; 41–60: moderate to severe burden; 61–88: severe burden). (18).

### Sample size calculation

Sample size was calculated using the following formula for cross-sectional studies:


$$\begin{array}{l} n={\left(\frac{Z_{1}-\alpha /2}{\delta }\right)}^{2}\times p\times \left(1-p\right) \end{array}$$

where n represents the required sample size; Z_1−α/2_ is the standard normal variate at 5% type I error (*p* < 0.05), which equals 1.96; *p* is the expected proportion in population (set at 0.5 to yield maximum sample size); and δ is the absolute error or precision (set at 0.05). Using these parameters, the minimum required sample size was calculated as 384. Considering a potential 10% non-response rate, the target sample size was increased to 480.

A total of 531 questionnaires were collected. After excluding invalid responses (1 refusal, 1 duplicate submission, 17 questionnaires completed in <90 s or more than 1,800 s, 51 cases where burn injury occurred more than 36 months ago, and 3 participants under 18 years of age), 459 valid questionnaires were included in the final analysis, yielding an effective response rate of 86.4%.

### Statistical analysis

Data analysis was performed using SPSS 22.0 (IBM, Armonk, NY, USA). Descriptive analysis of the demographic information and the scores across each dimension began with a normality test for the distribution of scores. If the data were normally distributed, they were presented as means and standard deviations (SD); if not, they were represented using medians along with the 25th and 75th percentiles. Categorical data were expressed as *n*(%). For comparing differences in scores among various demographic groups, continuous variables were analyzed using the *t*-test for normally distributed data between two groups, and the Wilcoxon Mann-Whitney test for non-normally distributed data. For three or more groups, if the continuous variables were normally distributed and had uniform variance, ANOVA was used; otherwise, the Kruskal-Wallis test was employed for non-normally distributed data. Correlation analysis for the scores in each dimension utilized the Pearson correlation coefficient if data distribution was normal; if not, the Spearman correlation coefficient was used. The scores from each dimension were treated as dependent variables in single-factor and multi-factor regression analyses to explore the relationship between demographic information and the scores. Results were categorized using the mean score for normally distributed data and the median score for non-normally distributed data. For the multivariate logistic regression analysis, we adopted an exploratory approach to comprehensively investigate all potential factors affecting the dependent variables. Variables with a *P*-value < 0.1 in univariate analyses were identified as potential influencing factors and included in the multivariate model. This approach allowed us to examine the independent associations of multiple variables simultaneously, rather than focusing on specific predetermined factors. For the practice dimension, the included variables were knowledge score, attitude score, caregiver burden, age, previous caregiving experience, cause of burns, and relationship with patient. For the caregiver burden analysis, age, residence, education level, income, previous caregiving experience, and patient age were included based on the same selection criteria. *P*-values were rounded to three decimal places, with *P* < 0.05 deemed statistically significant.

### Ethical considerations

This study was approved by the Ethic Committee of Taizhou Hospital in Zhejiang Province (KL20231013), and written informed consent was electronically obtained from all participants before the administration of questionnaires. The participants were informed about the study objectives, potential risks and benefits, and their right to withdraw at any time. Participation was entirely voluntary, and no monetary or material incentives were provided. Participants were recruited via hospital-based invitation during follow-up or inpatient care. All data were anonymized, and no personally identifiable information was collected. Responses were used solely for academic research purposes and were not shared beyond the research team.

## Result

The dataset comprised 459 valid cases. The overall reliability of the structured questionnaire was 0.848, with subscales for knowledge, attitude, and practice scoring 0.931, 0.694, and 0.789, respectively. The Kaiser-Meyer-Olkin (KMO) test yielded a value of 0.938 (*P* < 0.001), indicating excellent sampling adequacy. Confirmatory factor analysis (CFA) supported the three-domain structure of the questionnaire (knowledge, attitude, and practice). The model demonstrated good fit with the following indices: Root Mean Square Error of Approximation (RMSEA) = 0.051 (reference: <0.08), Standardized Root Mean Square Residual (SRMR) = 0.047 (reference: <0.08), Tucker-Lewis Index (TLI) = 0.925 (reference: >0.8), and Comparative Fit Index (CFI) = 0.931 (reference: >0.8). All items showed significant factor loadings (*P* < 0.001) on their respective domains, with standardized estimates ranging from 0.83 to 1.46 for knowledge items, 1.00 to 1.21 for attitude items, and 1.00 to 1.16 for practice items (as detailed in Supplementary Figure 1, Supplementary Tables 1, 2). The participant demographic included 253 females (55.12%), with an average age of 35.70 ± 9.45 years. Of these, 210 (45.75%) held at least a bachelor's degree, and 267 (58.17%) had cared for patients with severe burn injuries. Regarding the caregivers' own personal history, 259 (56.43%) reported having experienced a burn injury themselves at some point previously; this experience was distinct from the severe burn injury of the patient they were currently caring for according to the study's inclusion criteria. The main cause of injury of severe burn patients cared for by participants was thermal burn (370 cases, 80.61%), and most of them (52.29%) were burns within 6 months. The average age of these patients was 38.75 ± 19.62 years, with males constituting 282 (61.44%) of the sample. Median scores [25th percentile, 75th percentile] for knowledge, attitude, practice, and caregiver burden were 12 (9, 14), 17 (16, 19), 33 [31, 37], and 16 (10, 23), respectively. Notably, variations in knowledge scores were significantly associated with gender (*P* = 0.013), education level (*P* = 0.005), prior experience in caring for severely burned patients (*P* = 0.041), burn location (eyes, *P* = 0.003), primary caregiver status (*P* = 0.025), and relationship to the patient (*P* = 0.002). Attitude scores varied significantly based on gender (*P* = 0.003), income (*P* = 0.011), personal burn experience (*P* < 0.001), cause of burns (*P* < 0.001), burn location (eyes, *P* < 0.001; lower limbs, *P* = 0.015; other areas, *P* = 0.008), and relationship to the patient (*P* < 0.001). Practice scores also showed significant variability linked to gender (*P* = 0.024), educational attainment (*P* = 0.003), income (*P* = 0.015), cause of burns (*P* < 0.001), burn location (eyes, *P* < 0.001; arm, *P* = 0.040; lower limbs, *P* = 0.028), and patient relationship (*P* < 0.001). Caregiver burden scores varied significantly with marital status (*P* = 0.034), residence (*P* < 0.001), education (*P* = 0.018), age of the patient (*P* = 0.033), primary caregiver status (*P* < 0.001), and patient relationship (*P* < 0.001) (as shown in Table 1). Furthermore, analysis based on the time elapsed since the patient's burn injury (categorized as <6, 7–12, 13–24, and ≥25 months) revealed no statistically significant differences in caregiver KAP scores or burden levels across these groups (*P* > 0.05 for all comparisons, see Table 1).

**Table 1:** Basic information of participants and KAP score.

***N* = 459**	***N* (%)**	**Knowledge**		**Attitude**		**Practice**		**Caregiver burden**	
		**Med [p25, p75]**	***P***	**Med [p25, p75]**	***P***	**Med [p25, p75]**	***P***	**Med [p25, p75]**	***P***
**Total score**		12 [9, 14]		17 [16, 19]		33 [31, 37]		16 [10, 23]	
**Age**	35.70 ± 9.45								
**Age group**			0.927		0.343		0.168		0.155
≤44 years old	374 (81.48)	12 [9, 14]		17 [16, 19]		33 [31, 37]		16 [10, 23]	
45–59 years old	81 (17.65)	12 [10, 14]		17 [16, 19]		35 [32, 37]		13 [8, 21]	
≥60 years old	4 (0.87)	13 [8.5, 14]		17.5 [16.5, 19]		34.5 [32.5, 38]		11.5 [4.5, 19]	
**Gender**			**0.013**		**0.003**		**0.024**		0.431
Male	206 (44.88)	12 [8, 14]		17 [15, 18]		33 [30, 36]		16 [11, 23]	
Female	253 (55.12)	13 [9, 14]		17 [16, 19]		34 [31, 38]		15 [9, 23]	
**Marital status**			0.149		0.815		0.292		**0.034**
Other	119 (25.93)	12 [8, 14]		17 [16, 19]		34 [31, 38]		14 [8, 20]	
Married	340 (74.07)	12.5 [9, 14]		17 [16, 19]		33 [31, 36.5]		16 [10.5, 24]	
**Residence**			0.169		0.125		0.315		**<0.001**
City	299 (65.14)	13 [9, 14]		17 [16, 18]		33 [31, 36]		16 [12, 24]	
Non-urban	160 (34.86)	12 [9, 14]		17.5 [16, 20]		34 [31, 38]		14 [6, 21]	
**Education**			**0.005**		0.056		**0.003**		**0.018**
b. Junior high school and below	63 (13.73)	11 [9, 13]		17 [16, 19]		33 [29, 37]		12 [5, 21]	
c. Highschool/technical secondary school	75 (16.34)	11 [8, 14]		16 [15, 18]		32 [30, 34]		17 [12, 26]	
d. College degree	111 (24.18)	12 [9, 14]		17 [16, 19]		34 [32, 38]		16 [9, 23]	
e. Bachelor degree or above	210 (45.75)	13 [10, 14]		17 [16, 19]		34 [31, 37]		16 [10, 23]	
**Income**			0.199		**0.011**		**0.015**		0.158
<50,00	84 (18.3)	12 [9, 14]		18 [16, 20]		34 [31, 39]		14 [5, 22]	
5,001–10,000	184 (40.09)	12 [9, 14]		17 [15.5, 18]		32 [31, 36]		15 [11, 23.5]	
10,001–20,000	142 (30.94)	13 [9, 14]		17 [16, 19]		34 [32, 37]		16 [10, 23]	
>20,000	49 (10.68)	13 [8, 14]		17 [16, 19]		34 [31, 36]		16 [14, 23]	
**Burn experience**			0.222		**<0.001**		0.070		0.076
Yes	259 (56.43)	12 [9, 14]		17 [16, 18]		33 [31, 35]		17 [10, 24]	
No	200 (43.57)	13 [10, 14]		18 [16, 20]		34 [30, 39.5]		15 [9, 22]	
**Do you have previous experience caring for patients with severe burns?**			**0.041**		0.148		0.510		0.444
Yes	267 (58.17)	13 [9, 14]		17 [16, 18]		34 [31, 36]		16 [11, 23]	
No	192 (41.83)	12 [8, 14]		18 [15, 20]		33 [29, 39]		15 [8, 23]	
**Cause of burns in patients**			0.064		**<0.001**		**<0.001**		0.278
Thermal burns (fire, high temperature steam, liquid, etc.)	370 (80.61)	13 [9, 14]		17 [16, 19]		34 [31, 37]		16 [10, 23]	
Chemical burns	53 (11.55)	11 [7, 14]		16 [15, 17]		32 [30, 34]		14 [10, 23]	
Electrical burns	36 (7.84)	11 [9, 14]		16 [15, 17]		31.5 [29, 35]		15.5 [12.5, 28]	
**Patient's burned area**									
Face	142 (30.94)	12 [9, 14]	0.253	17 [16, 19]	0.849	32 [30, 37]	0.107	16 [10, 22]	0.886
Eye	78 (16.99)	10 [7, 14]	**0.003**	16 [14, 18]	**<0.001**	31 [29, 33]	**<0.001**	15.5 [10, 22]	0.971
Arm	330 (71.9)	12 [9, 14]	0.136	17 [16, 19]	0.436	34 [31, 37]	**0.040**	16 [10, 23]	0.322
Trunk	224 (48.8)	12 [9, 14]	0.740	17 [16, 19]	0.277	34 [31, 36]	0.609	16 [11, 24]	0.097
Lower limbs	124 (27.02)	13 [9, 14]	0.620	18 [16, 20]	**0.015**	34.5 [31, 39]	**0.028**	15 [8, 23.5]	0.563
Other	12 (2.61)	13 [12, 14]	0.220	19 [17.5, 20]	**0.008**	37.5 [34.5, 38.5]	0.052	8 [2.5, 19.5]	0.074
**Time since the patient was burned (months)**	9.55 ± 9.08								
**Time since the patient was burned (group)**			0.242		0.744		0.169		0.314
6 months and below	240 (52.29)	12 [8, 14]		17 [16, 19]		33 [30, 36]		16 [10, 23.5]	
July–December	122 (26.58)	13 [10, 14]		17 [16, 19]		34 [31, 38]		14 [8, 22]	
13–24 months	55 (11.98)	13 [10, 14]		17 [15, 19]		34 [31, 36]		15 [11, 22]	
25–36 months	42 (9.15)	13 [10, 14]		17 [16, 19]		33 [31, 37]		18.5 [13, 23]	
**Patient age (years)**	38.75 ± 19.62								
**Patient age (group)**			0.411		0.460		0.953		**0.033**
0–6 years old	22 (4.79)	11 [7, 14]		18 [16, 19]		35 [29, 37]		19.5 [12, 28]	
7–17 years old	46 (10.02)	12 [8, 14]		17 [15, 19]		33 [31, 36]		20 [12, 27]	
18–44 years old	200 (43.57)	13 [9, 14]		17 [16, 19]		34 [31, 37]		16 [10, 22]	
45–59 years old	128 (27.89)	12 [9, 14]		17 [16, 18]		33 [31, 36.5]		14 [9.5, 22]	
50 years and above	63 (13.73)	13 [9, 14]		18 [15, 20]		33 [31, 38]		14 [7, 23]	
**Patient gender**			0.204		0.187		0.439		0.131
Male	282 (61.44)	12 [9, 14]		17 [15, 19]		33 [31, 37]		16 [10, 24]	
Female	177 (38.56)	13 [9, 14]		17 [16, 19]		33 [31, 37]		15 [10, 22]	
**Are you the patient's primary caregiver?**			**0.025**		0.454		0.213		**<0.001**
Yes	296 (64.49)	12 [9, 14]		17 [15, 19]		34 [31, 37]		18 [12, 24]	
No	163 (35.51)	13 [9, 14]		17 [16, 19]		32 [31, 37]		12 [7, 19]	
**Relationship with patient**			**0.002**		**<0.001**		**<0.001**		**<0.001**
Parents	96 (20.92)	11 [8, 13]		16 [15, 18]		32 [29, 35]		20 [12, 25.5]	
Brothers and sisters	82 (17.86)	12.5 [10, 14]		17 [16, 19]		34 [31, 36]		15 [9, 20]	
Companion	61 (13.29)	12 [8, 14]		17 [16, 19]		34 [32, 37]		18 [13, 23]	
Other relationships (children/grandparents/other relationships)	220 (47.93)	13 [10, 14]		18 [16, 20]		34 [31, 40]		14 [9, 22]	

Bold values indicate statistical significance (*P* < 0.05).

**Figure 1:**
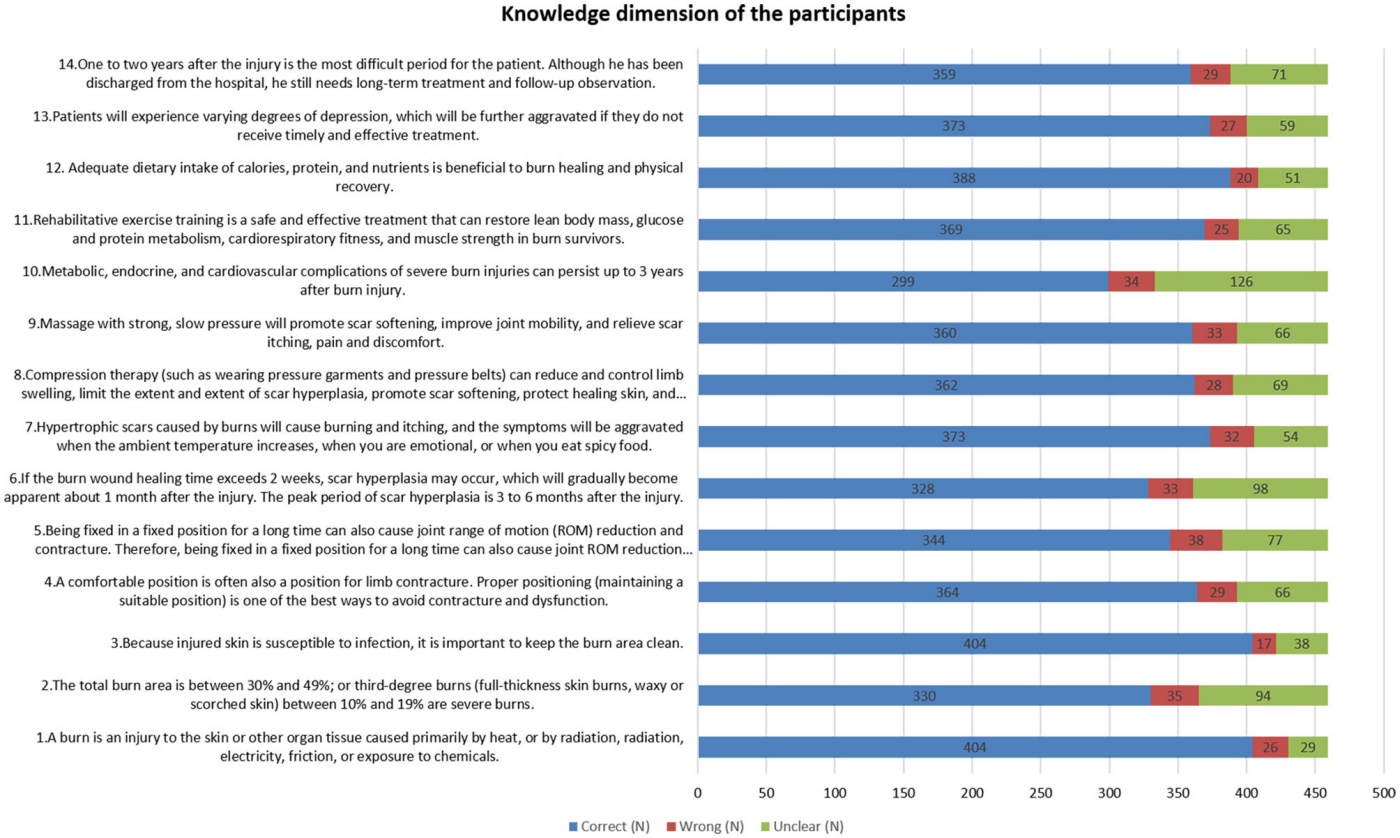
Knowledge dimension of the participants.

In the knowledge domain, the item with the highest correct response rate was related to the definition of burns, with 88.02% of participants identifying burns as injuries caused primarily by heat, radiation, electricity, friction, or chemical exposure (K1). Conversely, the item most often answered incorrectly involved the effects of prolonged immobility on joint range of motion and contractures (K5), with an error rate of 8.28%. The statement about long-term complications of severe burns being unclear was identified by 27.4% of participants (K10) (as detailed in Figure 1).

**Figure 2:**
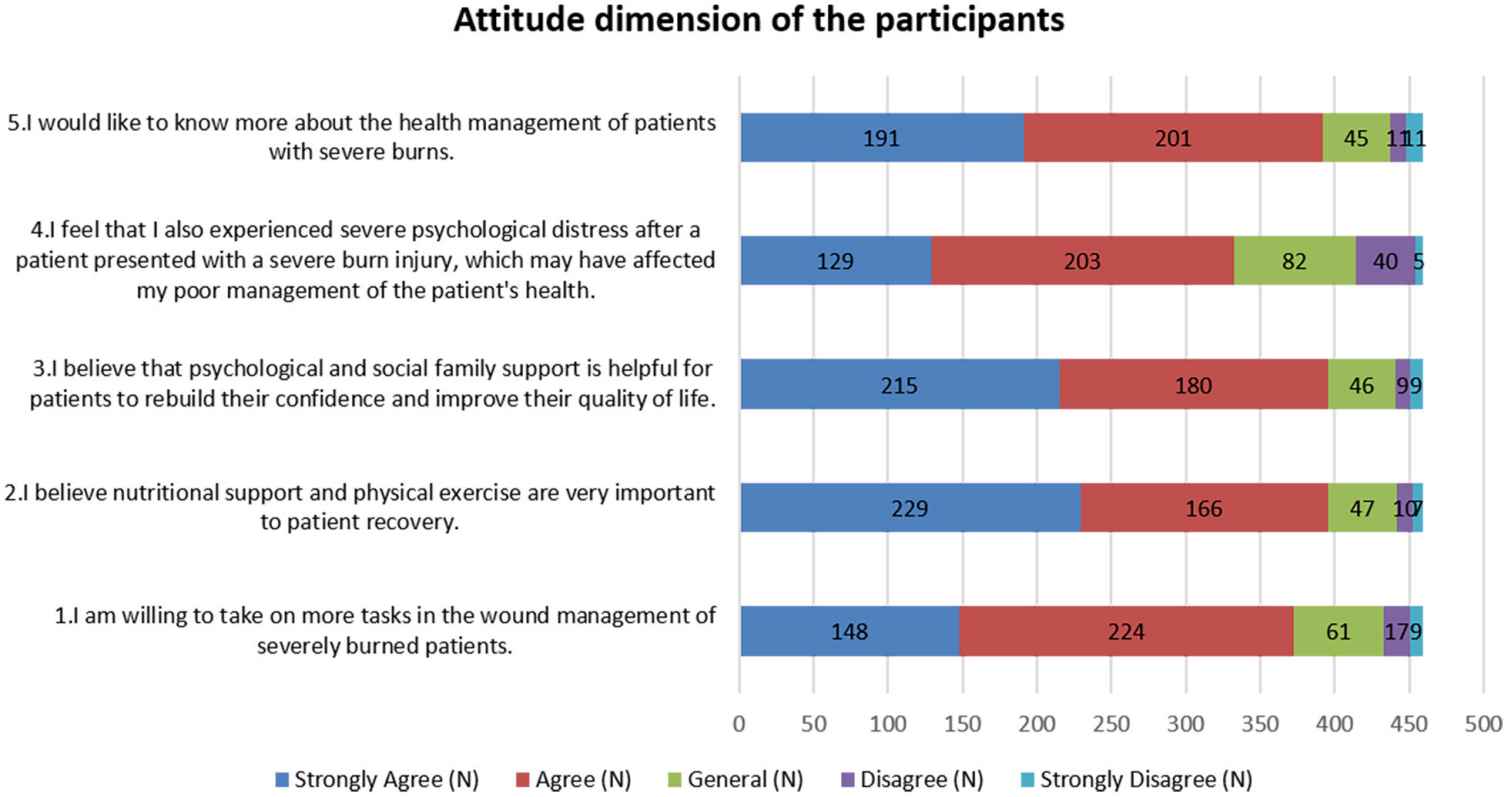
Attitude dimension of the participants.

Attitudes toward patient recovery were predominantly positive: 49.89% strongly agreed that nutritional support and physical activity were crucial (A2), 46.84% strongly felt that psychological and social support could significantly enhance patient confidence and quality of life (A3), and 48.8% were willing to undertake additional tasks in wound management for severely burned patients (A1). However, significant concern was noted regarding caregiver stress, with 28.1% strongly agreeing and 44.2% agreeing that caregivers suffer severe psychological distress, which could impair patient care (A4) (as shown in Figure 2).

**Figure 3:**
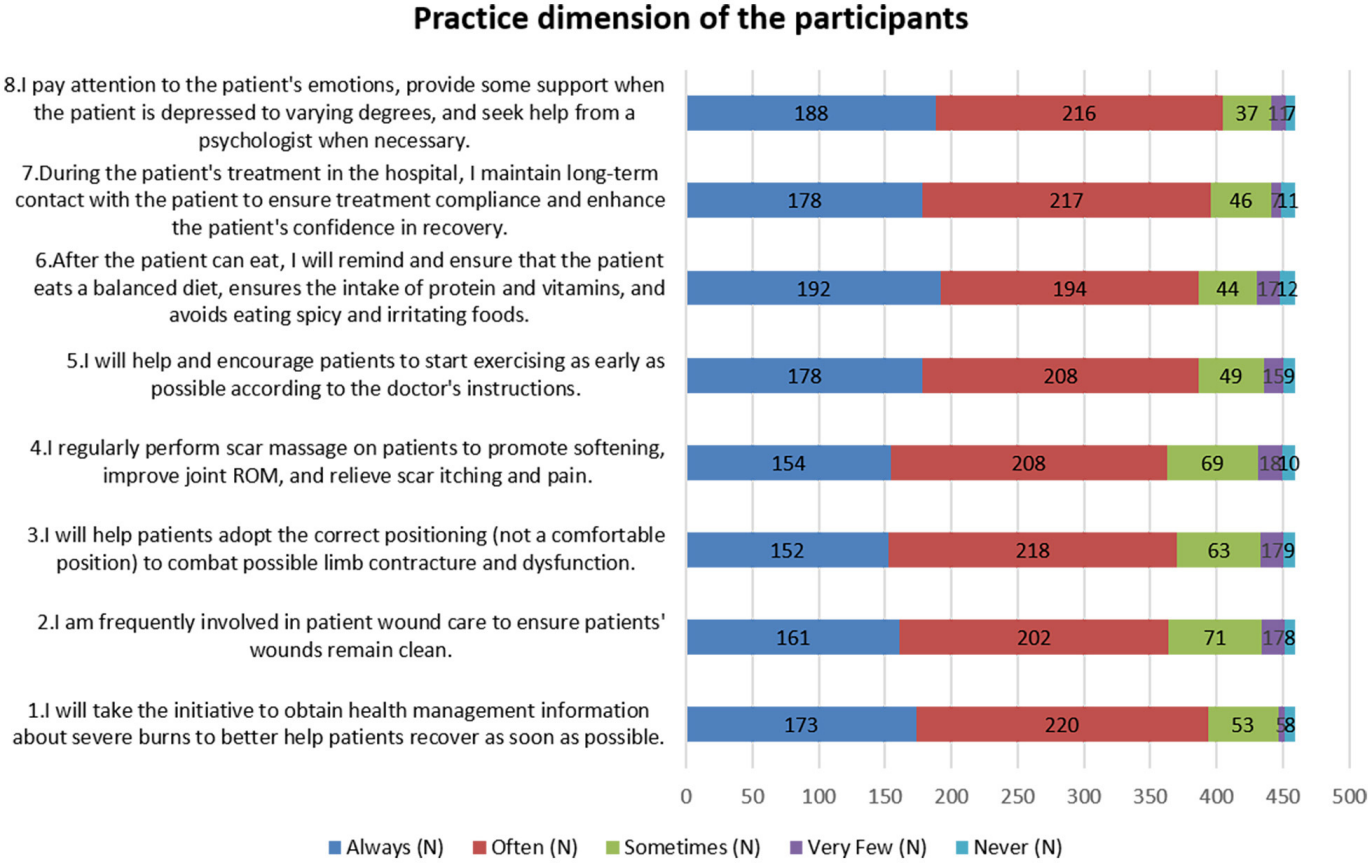
Practice dimension of the participants.

Practice responses indicated that 41.83% always reminded patients about dietary precautions once they could eat (P6), and 47.93% frequently sought information on managing severe burns (P1). However, involvement in wound care was sporadic, with 15.47% sometimes participating (P2), and 15.03% occasionally performing scar massages. Fewer than 10% rarely or never engaged in these practices (as shown in Figure 3).

Correlation analysis revealed significant relationships among the study variables: knowledge was correlated with attitude (*r* = 0.3517, *P* < 0.001), practice (*r* = 0.3642, *P* < 0.001), and caregiver burden (*r* = −0.1784, *P* < 0.001). Attitude was strongly correlated with practice (*r* = 0.7542, *P* < 0.001) and negatively correlated with caregiver burden (*r* = −0.2407, *P* < 0.001). Practice also demonstrated a negative correlation with caregiver burden (*r* = −0.2463, *P* < 0.001) (as presented in Table 2).

**Table 2:** Correlation analysis of KAP scores.

**Dimension**	**Knowledge dimension**	**Attitude**	**Practice**	**Caregiver burden**
**Knowledge dimension**	1			
Attitude	0.3517 (*P* < 0.001)	1		
Practice	0.3642 (*P* < 0.001)	0.7542 (*P* < 0.001)	1	
Caregiver burden	−0.1784 (*P* < 0.001)	−0.2407 (*P* < 0.001)	−0.2463 (*P* < 0.001)	1

**Figure 4:**
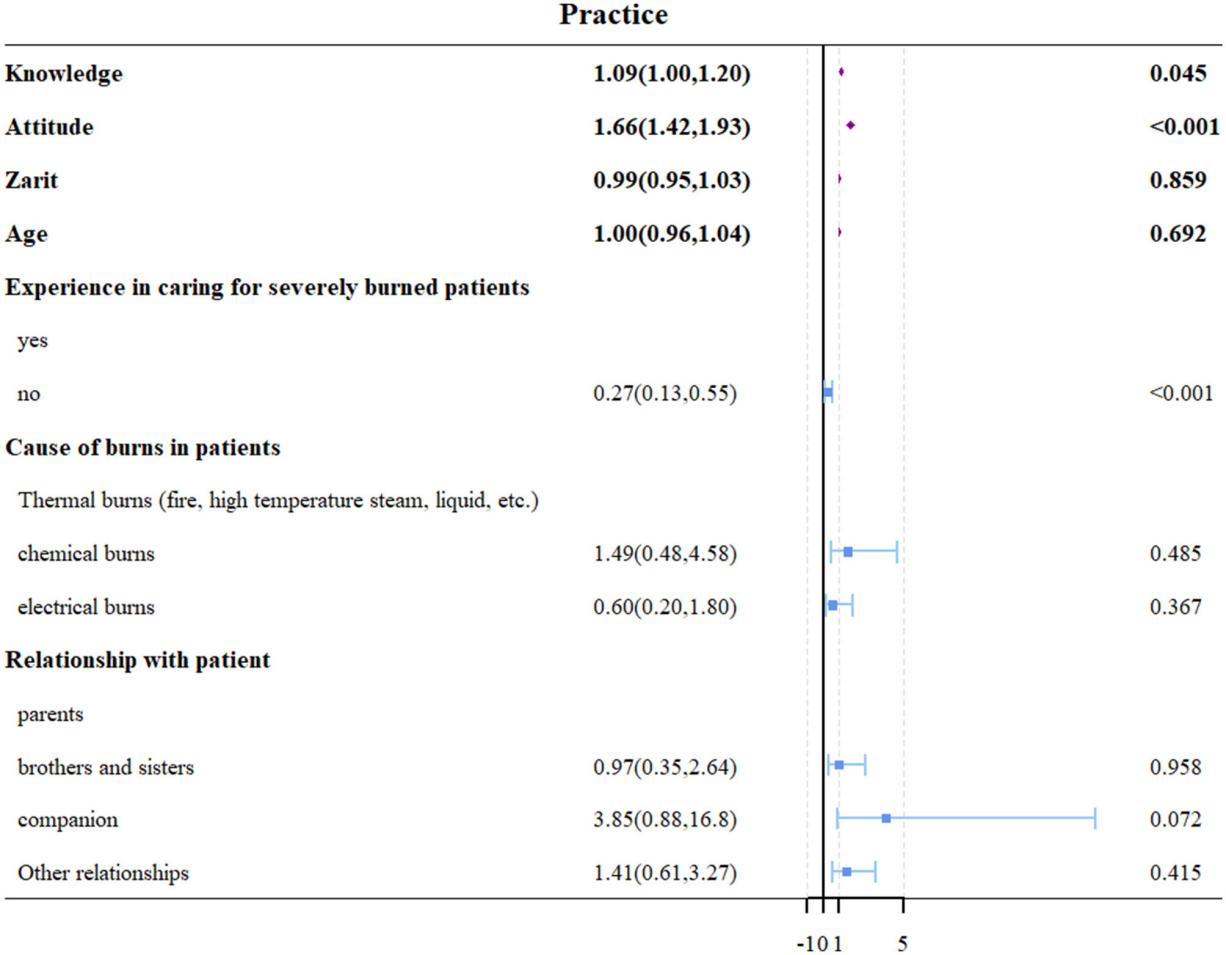
Forest plot of multivariate logistic regression analysis for factors associated with practice scores.

**Figure 5:**
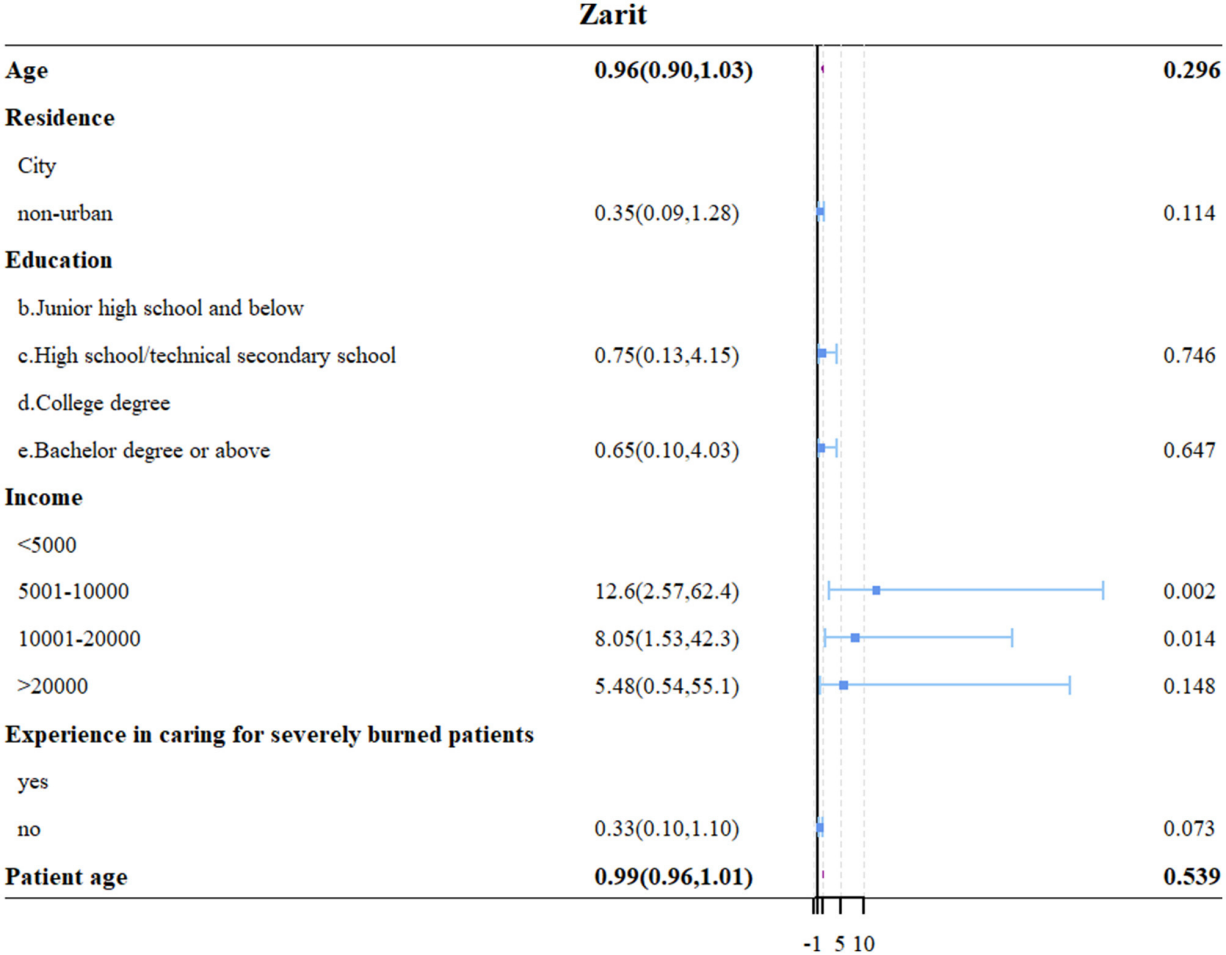
Forest plot of multivariate logistic regression analysis for factors associated with caregiver burden.

Multivariate logistic regression analysis identified that a higher knowledge score (OR = 1.09, 95% CI: [1.00, 1.209], *P* = 0.045), a higher attitude score (OR = 1.66, 95% CI: [1.42, 1.93], *P* < 0.001), and lack of previous experience caring for patients with severe burns (OR = 0.27, 95% CI: [0.13, 0.55], *P* < 0.001) were independently associated with better practice outcomes (Table 3, Figure 4). Additionally, income levels of 5,001–10,000 Yuan (OR = 12.6, 95% CI: [2.57, 62.4], *P* = 0.002) and 10,001–20,000 Yuan (OR = 8.05, 95% CI: [1.53, 42.3], *P* = 0.014) were found to be independently associated with increased caregiver burden (Table 4, Figure 5).

**Table 3:** Univariate and multivariate analysis for practice dimension.

**Practical dimension**	**Univariate**		**Multivariate**	
	**95%CI**	***P***	**95%CI**	***P***
**Knowledge dimension**	1.26 (1.17, 1.35)	**0**	1.09 (1.00, 1.20)	**0.045**
**Attitude dimension**	1.77 (1.54, 2.04)	**0**	1.66 (1.42, 1.93)	**0**
**Caregiver burden**	0.95 (0.93, 0.98)	**0.003**	0.99 (0.95, 1.03)	0.859
**Age**	1.03 (1.00, 1.06)	**0.017**	1.00 (0.96, 1.04)	0.692
**Do you have previous experience caring for patients with severe burns?**				
Yes				
No	0.27 (0.15, 0.47)	**<0.001**	0.27 (0.13, 0.55)	**0**
**Cause of burns in patients**				
**Thermal burns (fire, high temperature steam, liquid, etc.)**				
Chemical burns	0.81 (0.36, 1.84)	0.629	1.49 (0.48, 4.58)	0.485
Electrical burns	0.43 (0.19, 0.98)	**0.046**	0.60 (0.20, 1.80)	0.367
**Relationship with patient**				
Parents				
Brothers and sisters	1.53 (0.69, 3.36)	0.285	0.97 (0.35, 2.64)	0.958
Companion	5.08 (1.44, 17.9)	**0.011**	3.85 (0.88, 16.8)	0.072
Other relationships	1.73 (0.92, 3.24)	0.086	1.41 (0.61, 3.27)	0.415

Bold values indicate statistical significance (*P* < 0.05).

**Table 4:** Univariate and multivariate analysis for caregiver burden.

**Caregiver burden**	**Single factor**		**Multivariate**	
	**95%CI**	***P***	**95%CI**	***P***
**Age**	0.94 (0.90, 0.99)	**0.035**	0.96 (0.90, 1.03)	0.296
**Residence**				
City				
Non-urban	0.20 (0.07, 0.60)	**0.004**	0.35 (0.09, 1.28)	0.114
**Education**				
b. Junior high school and below				
c. High school/technical secondary school	3.49 (0.88, 13.7)	0.074	0.75 (0.13, 4.15)	0.746
d. College degree				
e. Bachelor degree or above	4.94 (1.64, 14.8)	**0.004**	0.65 (0.10, 4.03)	0.647
**Income**				
<5,000				
5,001–10,000	15.1 (3.31, 69.4)	**0**	12.6 (2.57, 62.4)	**0.002**
10,001–20,000	11.6 (2.54, 53.5)	**0.002**	8.05 (1.53, 42.3)	**0.014**
>20,000	7.99 (1.00, 63.5)	**0.049**	5.48 (0.54, 55.1)	0.148
**Do you have previous experience caring for patients with severe burns?**				
Yes				
No	0.28 (0.09, 0.82)	**0.021**	0.33 (0.10, 1.10)	0.073
**Patient age**	0.97 (0.94, 0.99)	**0.029**	0.99 (0.96, 1.01)	0.539

Bold values indicate statistical significance (*P* < 0.05).

## Discussion

Our findings indicate that: (1) Family members generally exhibit positive attitudes and proactive practices, but have suboptimal levels of knowledge; (2) Significant correlations exist among KAP dimensions, with knowledge positively associated with attitudes and practices; (3) Higher levels of knowledge, attitudes, and practices are associated with lower caregiver burden; (4) Demographic and clinical factors—including gender, education level, income, previous caregiving experience, and relationship to the patient—significantly influence KAP scores and caregiver burden. To enhance clinical practices in burn care, targeted educational interventions should be implemented to improve caregivers' knowledge, as higher knowledge levels correlate with better attitudes and more proactive practices. These results directly address our hypotheses, demonstrating the interdependence of KAP elements and their joint influence on caregiver burden. The evidence supports the theoretical premise of the KAP model, wherein knowledge serves as the basis for shaping attitudes, which in turn drive caregiving behaviors. Notably, all three KAP components were inversely correlated with caregiver burden, reinforcing the model's practical applicability in the context of severe burn care.

These findings align with several previous studies. A systematic review in Burns emphasized the critical role of multiplatform communication and support systems for caregivers of burn patients, highlighting the importance of accessible information and guidance (6). The relationship between caregiver knowledge and patient outcomes has been widely documented, noting that emotional preparedness and adequate training significantly impact care quality in burn units (25). The burden experienced by caregivers in our study mirrors findings from similar healthcare contexts. A study identified that e-mental health interventions could effectively support informal caregivers, suggesting the potential for digital platforms to enhance caregiver education and reduce burden (21). Our findings align with recent literature on caregiver experiences in burn care. A systematic review in Burns emphasized the critical role of multiplatform communication and support systems for caregivers of burn patients, highlighting the importance of accessible information and guidance (6). The relationship between caregiver knowledge and patient outcomes has been widely documented, noting that emotional preparedness and adequate training significantly impact care quality in burn units (25). The burden experienced by caregivers in our study mirrors findings from similar healthcare contexts. For instance, A study identified that e-mental health interventions could effectively support informal caregivers, suggesting the potential for digital platforms to enhance caregiver education and reduce burden (21). This is particularly relevant given our findings regarding the correlation between knowledge levels and caregiver burden.

Furthermore, recent research demonstrated the importance of first aid knowledge and ongoing education for burn care (26), while another study highlighted the effectiveness of simulation-based training in improving caregiver competence (3). Significantly, the study underscores the influence of socio-demographic factors on caregiving dynamics. Higher education levels were associated with better knowledge outcomes, a finding that is consistent with the literature suggesting that educational attainment enhances health literacy (27). Gender differences were also pronounced, with females exhibiting superior outcomes in knowledge, attitudes, and practices compared to males, a pattern observed in other caregiver studies (2). This gender disparity can be attributed to several sociocultural and behavioral factors. In traditional Chinese healthcare settings, women often assume primary caregiving responsibilities, leading to more accumulated care experience. A study found that female caregivers showed higher emotional engagement and attention to detail in burn care settings (25). Moreover, a study reported that women demonstrate greater proactivity in seeking health information and participating in healthcare management education (28). The emotional and practical involvement of female caregivers often translates into better knowledge retention and care practices, as evidenced by their higher participation rates in care-related training programs. Lower income levels, while associated with higher attitude scores, suggesting that individuals with fewer resources may develop stronger emotional commitments to caregiving despite facing higher stress and resource constraints. Middle-income caregivers may often maintain full-time employment while managing caregiving responsibilities, face higher societal expectations for providing quality care, and may be ineligible for financial assistance programs available to lower-income groups. Additionally, these caregivers might experience greater opportunity costs and work-related stress as they balance professional obligations with caregiving duties. Financial strain exacerbates caregiver stress, leading to significant emotional and physical tolls (29). Conversely, lack of previous caregiving experience is linked to poorer practice outcomes, possibly due to increased anxiety and uncertainty impairing caregiving abilities.

The correlation analysis in this study compellingly supports the interconnectedness of knowledge, attitudes, and practices among caregivers, underscoring a cycle where enhancing one positively influences the others. This aligns with previous study, suggesting that improved knowledge leads to better attitudes and more effective practices, thereby enhancing overall care quality (28). Notably, the analysis demonstrates that higher knowledge levels are associated with more favorable attitudes and more effective practices, reinforcing the hypothesis that informed caregivers can manage their roles more effectively. Additionally, the study reveals significant negative correlations between these variables and caregiver burden, indicating that as knowledge, attitudes, and practices improve, caregiver burden decreases. This suggests that caregivers who are better informed and maintain positive attitudes are likely to experience less stress and strain, which can contribute to a healthier caregiving environment.

In the knowledge dimension, a significant proportion of participants displayed a solid grasp of basic burn care principles, such as the importance of keeping burn areas clean and the relationship between burn severity and required care levels. However, there are noticeable gaps in more complex aspects, such as the long-term complications of severe burns and specifics of scar management. To improve knowledge, targeted educational interventions could be highly beneficial. For instance, creating detailed online modules that cover less understood topics like metabolic and cardiovascular complications post-burn could address specific knowledge deficits. Additionally, incorporating interactive elements such as quizzes and visual aids could enhance learning. Considering the popularity of social media in China, platforms like WeChat could be utilized to disseminate bite-sized educational content, which would make learning more accessible and engaging for caregivers (6, 30, 31).

The attitude dimension reveals a generally positive outlook toward the management of burn injuries, with a substantial majority acknowledging the importance of nutritional support and psychological care. However, a smaller yet significant number of participants felt overwhelmed by the psychological impact of managing severe burns, which could hinder effective patient care. This is reminiscent of findings, that caregiver stress negatively impacted care quality, highlighting the need for psychological support for caregivers themselves (21). Improvement suggestions here could include the development of support groups and stress management workshops, which could be offered both in-person and online to ensure wide accessibility. Facilitating regular workshops via WeChat groups, where caregivers can share experiences and coping strategies, might also reduce feelings of isolation and stress. Additionally, introducing routine psychological assessments for caregivers could help identify those in need of more intensive support early on, potentially preventing burnout (25, 32).

Regarding practice, while many participants are proactive in seeking information and managing patient care, there are gaps in consistent practice, especially in advanced care techniques such as scar management and dietary adjustments post-injury. To bridge this gap, practical training sessions could be invaluable. Hands-on workshops that allow caregivers to practice scar massage or the preparation of suitable meals for burn patients under professional supervision could boost confidence and competence. Additionally, creating video tutorials that can be accessed anytime on platforms would provide caregivers with the flexibility to learn and revisit techniques as needed. Another effective measure could be the implementation of mentorship programs, where experienced caregivers can guide less experienced ones, enhancing practical skills through peer learning and support (3, 26). In clinical settings, hospitals could establish integrated caregiver support frameworks that include structured discharge planning, caregiver competency assessments, and referral pathways to community support networks. These interventions could be initiated early during hospitalization and extended into post-discharge care to ensure continuity. Additionally, digital health tools, such as mobile apps or WeChat-based health education platforms, could be developed to deliver personalized learning modules and real-time support for caregivers, particularly in remote or resource-limited areas. Health education programs should emphasize not only medical aspects of burn care, but also coping strategies, stress reduction techniques, and communication skills to support caregiver resilience. Future research should adopt longitudinal designs to track the evolution of KAP and caregiver burden over time, particularly during different recovery stages of severe burns. Intervention studies are warranted to evaluate the effectiveness of targeted caregiver education programs on long-term care outcomes and burden reduction. Furthermore, qualitative studies exploring caregivers' lived experiences may provide deeper insight into emotional, cultural, and contextual factors that quantitative data may overlook. Tailoring interventions based on gender, educational background, and prior caregiving experience may improve their relevance and uptake.

This study has several limitations. First, the cross-sectional design limits the ability to infer causality between knowledge, attitudes, practices, and caregiver burden. Longitudinal or intervention-based studies are needed to determine the directionality of these relationships over time. Second, while the KAP model provided a useful framework for exploring caregiver competencies, it may oversimplify the complex psychological and contextual factors influencing caregiver behavior, such as emotional coping, cultural expectations, or family dynamics. Third, the use of convenience sampling in a single province restricts the generalizability of the findings, especially to rural or less-developed regions. Fourth, the reliance on self-reported measures may introduce social desirability or recall bias, potentially affecting the accuracy of reported practices and perceived burden. Fifth, although the questionnaire demonstrated good reliability and construct validity, it may not fully capture the evolving and multifaceted nature of burn care knowledge, especially regarding long-term management. Future studies should incorporate mixed-methods approaches, recruit more diverse populations, and explore additional theoretical models that capture the emotional and social dimensions of caregiving.

## Conclusion

In conclusion, the study demonstrates that while family members generally hold positive attitudes and engage in proactive practices toward managing severe burn injuries, there is a notable deficiency in knowledge, which correlates with variations in practice outcomes and caregiver burden. It is recommended that healthcare providers implement targeted educational programs to enhance the knowledge base of family caregivers, which could potentially improve care practices and alleviate caregiver burden. Specifically, structured caregiver education programs focusing on long-term burn complications and psychological support should be implemented through various formats, including interactive online modules, in-person workshops, and ongoing support groups. These programs should emphasize both technical care skills and emotional coping strategies to comprehensively address the identified knowledge gaps and support needs. To further alleviate caregiver burden, healthcare institutions should establish peer support networks for experience sharing, provide regular psychological counseling services, and implement respite care programs that allow caregivers temporary breaks from their duties. This comprehensive support system could help prevent caregiver burnout while maintaining quality patient care. For clinical implementation, hospitals should integrate caregiver education into the standard burn care protocol, starting with systematic training during hospitalization and followed by competency assessments before discharge. A structured discharge planning process should include take-home instructions and ensure family members' access to ongoing support resources.

## Data Availability

The original contributions presented in the study are included in the article/Supplementary material, further inquiries can be directed to the corresponding author.

## Supplementary material

Supplementary Figure 1 Path diagram of confirmatory factor analysis for the KAP questionnaire.

Supplementary Table 1 Model fit indices of confirmatory factor analysis.

## References

[1] Bookman A Harrington M. Family caregivers: a shadow workforce in the geriatric health care system? J Health Polit Policy Law. (2007) 32:1005–41. 10.1215/03616878-2007-040 18000158

[2] Wang N Chen Y Ren B Xiang Y Zhao N Zhan X. A cross-sectional study: comparison of public perceptions of adverse drug reaction reporting and monitoring in Eastern and Western China. BMC Health Serv Res. (2022) 22:318. 10.1186/s12913-022-07720-0 35260158 PMC8905784

[3] Farzaneh SB Devetzis K Kamyab AA Sousi S Zargaran A Zargaran D. Emergency burn education: evaluating a surgical simulation-based intervention. J Plast Reconstr Aesthet Surg. (2023) 82:137–40. 10.1016/j.bjps.2023.03.001 37167714

[4] Von Elm E Altman DG Egger M Pocock SJ Gøtzsche PC Vandenbroucke JP. The strengthening the reporting of observational studies in epidemiology (strobe) statement: guidelines for reporting observational studies. Int J Surg. (2014) 12:1495–9. 10.1016/j.ijsu.2014.07.013 25046131

[5] Selman LE Brighton LJ Sinclair S Karvinen I Egan R Speck P. Patients' and caregivers' needs, experiences, preferences and research priorities in spiritual care: a focus group study across nine countries. Palliat Med. (2018) 32:216–30. 10.1177/0269216317734954 29020846 PMC5758929

[6] Mc Kittrick A Kornhaber R de Jong A Allorto N Vana LPM Chong SJ. The role of multiplatform messaging applications in burns care and rehabilitation: a systematic review. Burns. (2024) 50:1424–36. 10.1016/j.burns.2024.03.013 38580579

[7] Kruczek C Kottapalli KR Dissanaike S Dzvova N Griswold JA Colmer-Hamood JA. Major transcriptome changes accompany the growth of pseudomonas aeruginosa in blood from patients with severe thermal injuries. PLoS ONE. (2016) 11:e0149229. 10.1371/journal.pone.0149229 26933952 PMC4774932

[8] Tejiram S Romanowski KS Palmieri TL. Initial management of severe burn injury. Curr Opin Crit Care. (2019) 25:647–52. 10.1097/MCC.0000000000000662 31567292

[9] Bergquist M Hästbacka J Glaumann C Freden F Huss F Lipcsey M. The time-course of the inflammatory response to major burn injury and its relation to organ failure and outcome. Burns. (2019) 45:354–63. 10.1016/j.burns.2018.09.001 30274808

[10] Dahiya P. Burns as a model of sirs. FBL. (2009) 14:4962–7. 10.2741/3580 19482598

[11] Mulder PPG Vlig M Fasse E Stoop MM Pijpe A van Zuijlen PPM. Burn-injured skin is marked by a prolonged local acute inflammatory response of innate immune cells and pro-inflammatory cytokines. Front Immunol. (2022) 13:4420. 10.3389/fimmu.2022.1034420 36451819 PMC9703075

[12] Wang L Yao Q Zhang YP Xia YL Gu Y Zhou HC. Systematic evaluation of qualitative research on the real experience of burn patients during rehabilitation. Zhonghua Shao Shang Yu Chuang Mian Xiu Fu Za Zhi. (2022) 38:69–76. 10.3760/cma.j.cn501120-20201130-00507 34839598 PMC11705272

[13] Maudet L Pasquier M Pantet O Albrecht R Carron PN. Prehospital management of burns requiring specialized burn centre evaluation: a single physician-based emergency medical service experience. Scand J Trauma Resusc Emerg Med. (2020) 28:84. 10.1186/s13049-020-00771-4 32819398 PMC7439538

[14] Snell JA Loh NH Mahambrey T Shokrollahi K. Clinical review: the critical care management of the burn patient. Crit Care. (2013) 17:241. 10.1186/cc12706 24093225 PMC4057496

[15] Spatuzzi R Giulietti MV Ricciuti M Merico F Meloni C Fabbietti P. Quality of life and burden in family caregivers of patients with advanced cancer in active treatment settings and hospice care: a comparative study. Death Stud. (2017) 41:276–83. 10.1080/07481187.2016.1273277 27982741

[16] Schulz R Sherwood PR. Physical and mental health effects of family caregiving. Am J Nurs. (2008) 108:23–7. 10.1097/01.NAJ.0000336406.45248.4c 18797217 PMC2791523

[17] Gao L Su S Du N Han Y Wei J Cao M. Medical and non-medical students' knowledge, attitude and willingness towards the Covid-19 vaccine in China: a cross-sectional online survey. Hum Vaccin Immunother. (2022) 18:2073757. 10.1080/21645515.2022.2073757 35612817 PMC9359383

[18] Ballesteros J Santos B González-Fraile E Muñoz-Hermoso P Domínguez-Panchón AI Martín-Carrasco M. Unidimensional 12-item zarit caregiver burden interview for the assessment of dementia caregivers' burden obtained by item response theory. Value Health. (2012) 15:1141–7. 10.1016/j.jval.2012.07.005 23244818

[19] Twinamasiko N Olum R Gwokyalya AM Nakityo I Wasswa E Sserunjogi E. Assessing knowledge, attitudes and practices towards Covid-19 public health preventive measures among patients at Mulago National Referral Hospital. Risk Manag Healthc Policy. (2021) 14:221–30. 10.2147/RMHP.S287379 33505175 PMC7829119

[20] Wang J Chen L Yu M He J. Impact of knowledge, attitude, and practice (Kap)-based rehabilitation education on the kap of patients with intervertebral disc herniation. Ann Palliat Med. (2020) 9:388–93. 10.21037/apm.2020.03.01 32233633

[21] Coumoundouros C von Essen L Sanderman R Woodford J. Implementation of E-mental health interventions for informal caregivers of adults with chronic diseases: a protocol for a mixed-methods systematic review with a qualitative comparative analysis. BMJ Open. (2020) 10:e035406. 10.1136/bmjopen-2019-035406 32565461 PMC7307546

[22] Palackic A Suman OE Porter C Murton AJ Crandall CG Rivas E. Rehabilitative exercise training for burn injury. Sports Med. (2021) 51:2469–82. 10.1007/s40279-021-01528-4 34339042 PMC8595583

[23] Chinese Association of Plastic and Aesthetic Medicine SMB. National expert consensus on early treatment of scars (2020 edition). Chin J Burns. (2021) 2:113–25. 10.3760/cma.j.cn501120-20200609-00300 33498097

[24] Lee F Suryohusodo AA. Knowledge, attitude, and practice assessment toward Covid-19 among communities in East Nusa Tenggara, Indonesia: a cross-sectional study. Front Public Health. (2022) 10:957630. 10.3389/fpubh.2022.957630 36388283 PMC9659730

[25] Caminati G Cappelli L Ferri P Artioli G Spadola M Spadola M. Emotional impact of clinical practice in burns unit among nursing students: a qualitative study. Acta Biomed. (2021) 92:e2021008. 10.23750/abm.v92iS2.11411 33855986 PMC8138809

[26] Alhusayni MA Alotaibi NM Alshaer AA Alnefaie A Alotaibi MM Albogami ARR. Assessment of awareness and practices related to burn injury first aid among the general public: cross-sectional study in Taif, Saudi Arabia. Cureus. (2023) 15:e45912. 10.7759/cureus.45912 37885549 PMC10599189

[27] Ligita T Wicking K Francis K Harvey N Nurjannah I. How people living with diabetes in indonesia learn about their disease: a grounded theory study. PLoS ONE. (2019) 14:e0212019. 10.1371/journal.pone.0212019 30794570 PMC6386238

[28] Ghalavand H Panahi S Sedghi S. Opportunities and challenges of social media for health knowledge management: a narrative review. J Educ Health Promot. (2020) 9:144. 10.4103/jehp.jehp_754_19 32766329 PMC7377150

[29] Tong H Qiu F Fan L. Characterising common challenges faced by parental caregivers of children with type 1 diabetes mellitus in Mainland China: a qualitative study. BMJ Open. (2022) 12:e048763. 10.1136/bmjopen-2021-048763 35017233 PMC8753393

[30] GBD 2021 Risk Factors Collaborators. Global Burden and Strength of Evidence for 88 Risk Factors in 204 Countries and 811 Subnational Locations, 1990-2021: A Systematic Analysis for the Global Burden of Disease Study 2021. Lancet. (2024) 403:2162–203. 10.1016/S0140-6736(24)00933-4 38762324 PMC11120204

[31] Phelps TB Archer AD Leonard M Collins H Burns JB Jr. Outcome of seatbelt education and safety program among teenagers. Am Surg. (2024) 90:1931–3. 10.1177/00031348241241744 38523078

[32] Khanduja K Bould MD Andrews M LeBlanc V Schebesta K Burns JK. Impact of unexpected death in a simulation scenario on skill retention, stress, and emotions: a simulation-based randomized controlled trial. Cureus. (2023) 15:e39715. 10.7759/cureus.39715 37398706 PMC10309656

