# Supplementary material for: Family members' knowledge, attitudes, practices, and caregiver burden in managing the health of patients with severe burn injuries
Source: Front Public Health. 2025 May 19;13:1450356. doi: 10.3389/fpubh.2025.1450356 (PMC12127349; doi:10.3389/fpubh.2025.1450356)
Supplement: Supplementary file 1 [file Table_1.doc]

Questionnaire number:

| Dear friends:  We are scientific researchers at ** Hospital and sincerely invite you to participate in our research. This study aims to understand family members’ understanding, attitude and practice of health management of patients with severe burns, and provide a basis for formulating scientific early intervention strategies, which may help more people and improve the health of patients in the future. Your participation in this study is voluntary, please see the instructions below if you agree to participate in this study.  1. Please complete the questionnaire. There is no right or wrong answer. You only need to fill it in based on the actual situation. You can ask us any questions during the answering process, and please submit them in time after completion.  2. This research is a simple questionnaire survey that will not cause harm to your physical and mental conditions, but will involve some privacy issues, such as your gender, age, etc. We will keep it strictly confidential and will not disclose your information. Please Feel free to fill it out.  3. As a participant, you can keep informed of the information and research progress related to this study at any time. If you decide to withdraw from the study, please inform us and your data will not be included in the research results.  Finally, we sincerely thank you for taking time out of your busy schedule to support our scientific research!  ⬜I acknowledge and consent to the use of collected data for scientific research.  Informed consent signature:  Participation time: Years Months Days |
| --- |

| **Part One Basic Information** | |
| --- | --- |
| You in the following information refer to the patient’s family members, that is, the person who filled out the questionnaire. | |
| 1. **your age:** | |
| 1. **Your gender:**a. Male b. Female |  |
| 1. **your marital status** | a.Unmarried  b.Married  c.Divorced  d.Widowed |
| 1. **Where you live:** | a.City  b.rural area |
| 1. **Your education level:** | a.Elementary school and below  b.Junior high school  c.High school/technical secondary school  d.College degree  e.Bachelor’s degree  f.Master’s degree and above |
| 1. **Your average monthly household income: (yuan)** | a.<5000  b.5000-10000  c.10001-20000  d.>20000 |
| 1. **Have you ever experienced a burn injury?** | a.Yes  b.No |
| 1. **Do you have previous experience caring for patients with severe burns:** | a.Yes  b.No |
| The following information is patient information |  |
| 1. **Cause of patient’s burns:** | 1. Thermal burns (fire, high temperature steam, liquid, etc.) 2. chemical burns 3. electrical burns |
| 1. **Patient’s burn site (multiple choices):** | 1. face 2. Eye 3. arm 4. trunk 5. other______ |
| 1. **Time since the patient was burned** | month |
| 1. **Patient age:** | year old |
| 1. **Patient gender:** | a. Man  b. Female |
| 1. **Are you the patient's primary caregiver?** | a.Yes  b.No |
| 1. **You are a patient of:** | a.Parents  b.grandparents  c. Brothers and sisters  d.Partner  e.Other relationships _ |

| **Part 2 Zarit Caregiver Burden Scale 12-item short form** | |
| --- | --- |
| The following questions reflect how you feel when caring for patients: Have you experienced any of the following feelings in the past week? Please read each of the following questions carefully and select the option that best applies to you. | |
| 1. Do you think you are short on time by caring for patients? | A. No  B. Occasionally  C． sometimes  D. often  E. always |
| 2. Do you think you feel stressed between taking care of your patients and trying to do your housework and work well? | A. No  B. Occasionally  C． sometimes  D. often  E. always |
| 3. Do you think the patient is dependent on you? | A. No  B. Occasionally  C． sometimes  D. often  E. always |
| 4. Do you feel nervous when patients are around you? | A. No  B. Occasionally  C． sometimes  D. often  E. always |
| 5. Do you feel that your health has been affected as a result of caring for patients? | A. No  B. Occasionally  C． sometimes  D. often  E. always |
| 6. Do you think that because you care for patients, you don’t have time to do your own personal things? | A. No  B. Occasionally  C． sometimes  D. often  E. always |
| 7. Do you feel that your social life is affected as a result of caring for patients? | A. No  B. Occasionally  C． sometimes  D. often  E. always |
| 8. Do you think it is possible for you to spend more time caring for patients? | A. No  B. Occasionally  C． sometimes  D. often  E. always |
| 9. Do you think that since you started nursing, it has become impossible to live according to your own wishes? | A. No  B. Occasionally  C． sometimes  D. often  E. always |
| 10. Do you hope that you can leave the patient to others to take care of you? | A. No  B. Occasionally  C． sometimes  D. often  E. always |
| 11. Have you ever had a situation where you didn’t know what to do with a patient? | A. No  B. Occasionally  C． sometimes  D. often  E. always |
| 12. Overall, how do you evaluate your nursing burden? | A. No  B. Occasionally  C． sometimes  D. often  E. always |

| **Part 3: Understanding of health management knowledge of patients with severe burns** | |
| --- | --- |
| Please decide whether the following description is true or false. If you cannot decide whether it is true or false, you can choose the unclear option. | |
| 1. **A burn is an injury to the skin or other organ tissue caused primarily by heat, or by radiation, radiation, electricity, friction, or exposure to chemicals.** | A. Correct, B. Wrong, C. Unclear |
| 1. **The total burn area is between 30% and 49%; or third-degree burns (full-thickness skin burns, waxy or scorched skin) between 10% and 19% are severe burns.** | A. Correct, B. Wrong, C. Unclear |
| 1. **Because injured skin is susceptible to infection, it is important to keep the burn area clean.** | A. Correct, B. Wrong, C. Unclear |
| 1. **A comfortable position is often also a position for limb contracture. Proper positioning (maintaining a suitable position) is one of the best ways to avoid contracture and dysfunction.** | A. Correct, B. Wrong, C. Unclear |
| 1. **Being fixed in a fixed position for a long time can also cause joint range of motion (ROM) reduction and contracture. Therefore, being fixed in a fixed position for a long time can also cause joint ROM reduction and contracture.** | A. Correct, B. Wrong, C. Unclear |
| 1. **If the burn wound healing time exceeds 2 weeks, scar hyperplasia may occur, which will gradually become apparent about 1 month after the injury. The peak period of scar hyperplasia is 3 to 6 months after the injury.** | A. Correct, B. Wrong, C. Unclear |
| 1. **Hypertrophic scars caused by burns will cause burning and itching, and the symptoms will be aggravated when the ambient temperature increases, when you are emotional, or when you eat spicy food.** | A. Correct, B. Wrong, C. Unclear |
| 1. **Compression therapy (such as wearing pressure garments and pressure belts) can reduce and control limb swelling, limit the extent and extent of scar hyperplasia, promote scar softening, protect healing skin, and reduce itching and pain.** | A. Correct, B. Wrong, C. Unclear |
| 1. **Massage with strong, slow pressure will promote scar softening, improve joint mobility, and relieve scar itching, pain and discomfort.** | A. Correct, B. Wrong, C. Unclear |
| 1. **Metabolic, endocrine, and cardiovascular complications of severe burn injuries can persist up to 3 years after burn injury.** | A. Correct, B. Wrong, C. Unclear |
| 1. **Rehabilitative exercise training is a safe and effective treatment that can restore lean body mass, glucose and protein metabolism, cardiorespiratory fitness, and muscle strength in burn survivors.** | A. Correct, B. Wrong, C. Unclear |
| 1. **Adequate dietary intake of calories, protein, and nutrients is beneficial to burn healing and physical recovery.** | A. Correct, B. Wrong, C. Unclear |
| 1. **Patients will experience varying degrees of depression, which will be further aggravated if they do not receive timely and effective treatment.** | A. Correct, B. Wrong, C. Unclear |
| 1. **One to two years after the injury is the most difficult period for the patient. Although he has been discharged from the hospital, he still needs long-term treatment and follow-up observation.** | A. Correct, B. Wrong, C. Unclear |

| **Part 4: Attitudes and Thoughts on Health Management of Severe Burn Patients** | |
| --- | --- |
| 1. **I am willing to take on more tasks in the wound management of severely burned patients.** | A. Strongly agree  B.Agree  C.General agree  D. Disagree  E.Strongly disagree |
| 1. **I believe nutritional support and physical exercise are very important to patient recovery.** | A. Strongly agree  B.Agree  C.General agree  D. Disagree  E.Strongly disagree |
| 1. **I believe that psychological and social family support is helpful for patients to rebuild their confidence and improve their quality of life.** | A. Strongly agree  B.Agree  C.General agree  D. Disagree  E.Strongly disagree |
| 1. **I feel that I also experienced severe psychological distress after a patient presented with a severe burn injury, which may have affected my poor management of the patient's health.** | A. Strongly agree  B.Agree  C.General agree  D. Disagree  E.Strongly disagree |
| 1. **I would like to know more about the health management of patients with severe burns.** | A. Strongly agree  B.Agree  C.General agree  D. Disagree  E.Strongly disagree |

| **Part 5: Behavioral practices for health management of patients with severe burns** | |
| --- | --- |
| 1. **I will take the initiative to obtain health management information about severe burns to better help patients recover as soon as possible.** | A. Very consistent  B.Conform to  C.General  D.Does not meet  E. Very inconsistent |
| 1. **I am frequently involved in patient wound care to ensure patients' wounds remain clean.** | A. Very consistent  B.Conform to  C.General  D.Does not meet  E. Very inconsistent |
| 1. **I will help patients adopt the correct positioning (not a comfortable position) to combat possible limb contracture and dysfunction.** | A. Very consistent  B.Conform to  C.General  D.Does not meet  E. Very inconsistent |
| 1. **I regularly perform scar massage on patients to promote softening, improve joint ROM, and relieve scar itching and pain.** | A. Very consistent  B.Conform to  C.General  D.Does not meet  E. Very inconsistent |
| 1. **I will help and encourage patients to start exercising as early as possible according to the doctor's instructions.** | A. Very consistent  B.Conform to  C.General  D.Does not meet  E. Very inconsistent |
| 1. **After the patient can eat, I will remind and ensure that the patient eats a balanced diet, ensures the intake of protein and vitamins, and avoids eating spicy and irritating foods.** | A. Very consistent  B.Conform to  C.General  D.Does not meet  E. Very inconsistent |
| 1. **During the patient's treatment in the hospital, I maintain long-term contact with the patient to ensure treatment compliance and enhance the patient's confidence in recovery.** | A. Very consistent  B.Conform to  C.General  D.Does not meet  E. Very inconsistent |
| 1. **I pay attention to the patient's emotions, provide some support when the patient is depressed to varying degrees, and seek help from a psychologist when necessary.** | A. Very consistent  B.Conform to  C.General  D.Does not meet  E. Very inconsistent |
