# Supplementary figures and images for: Family members' knowledge, attitudes, practices, and caregiver burden in managing the health of patients with severe burn injuries
Source: Front Public Health. 2025 May 19;13:1450356. doi: 10.3389/fpubh.2025.1450356 (PMC12127349; doi:10.3389/fpubh.2025.1450356)

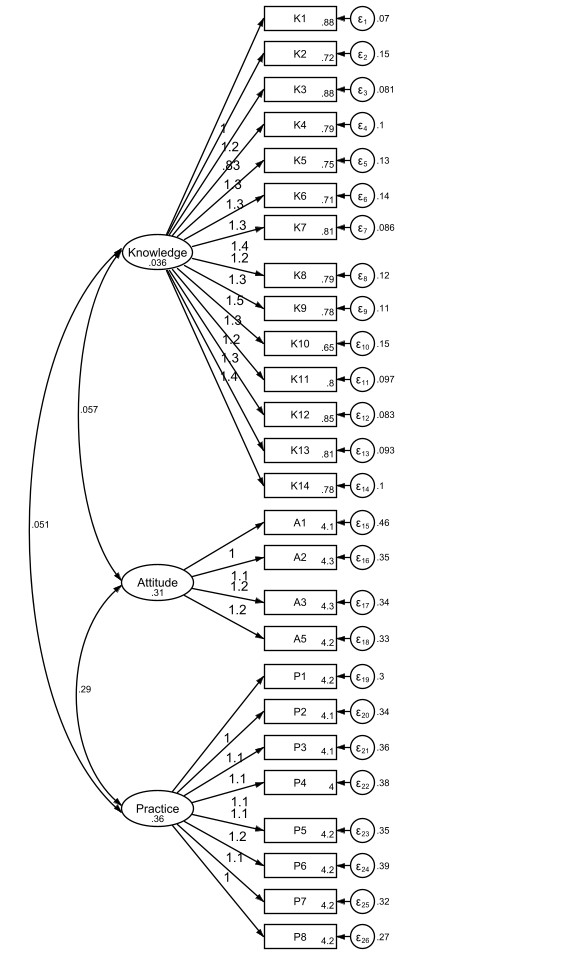

Supplement: Supplementary Figure 1 — Path diagram of confirmatory factor analysis for the KAP questionnaire. [file Image_1.jpeg]
