# Supplementary material for: Family members' knowledge, attitudes, practices, and caregiver burden in managing the health of patients with severe burn injuries
Source: Front Public Health. 2025 May 19;13:1450356. doi: 10.3389/fpubh.2025.1450356 (PMC12127349; doi:10.3389/fpubh.2025.1450356)
Supplement: Supplementary Table 1 — Model fit indices of confirmatory factor analysis. [file Table_2.docx]

**Supplementary Table 1. Model fit indices of confirmatory factor analysis**

| Indicators | Reference | Results |
| --- | --- | --- |
| RMSEA | <0.08 Good | 0.051 |
| SRMR | <0.08 Good | 0.047 |
| TLI | >0.8 Good | 0.925 |
| CFI | >0.8 Good | 0.931 |

**Supplementary Table 2. Standardized factor loadings from confirmatory factor analysis**

|  |  | Estimate | P>\|z\| |
| --- | --- | --- | --- |
| K1 | Knowledge | 1 |  |
| K2 | Knowledge | 1.24 | <0.001 |
| K3 | Knowledge | 0.83 | <0.001 |
| K4 | Knowledge | 1.32 | <0.001 |
| K5 | Knowledge | 1.31 | <0.001 |
| K6 | Knowledge | 1.29 | <0.001 |
| K7 | Knowledge | 1.36 | <0.001 |
| K8 | Knowledge | 1.19 | <0.001 |
| K9 | Knowledge | 1.33 | <0.001 |
| K10 | Knowledge | 1.46 | <0.001 |
| K11 | Knowledge | 1.30 | <0.001 |
| K12 | Knowledge | 1.15 | <0.001 |
| K13 | Knowledge | 1.28 | <0.001 |
| K14 | Knowledge | 1.37 | <0.001 |
| A1 | Attitude | 1 |  |
| A2 | Attitude | 1.11 | <0.001 |
| A3 | Attitude | 1.15 | <0.001 |
| A4 | Attitude | 1.21 | <0.001 |
| P1 | Practice | 1 |  |
| P2 | Practice | 1.14 | <0.001 |
| P3 | Practice | 1.09 | <0.001 |
| P4 | Practice | 1.13 | <0.001 |
| P5 | Practice | 1.09 | <0.001 |
| P6 | Practice | 1.16 | <0.001 |
| P7 | Practice | 1.07 | <0.001 |
| P8 | Practice | 1.05 | <0.001 |
